# Microbial Cryptotopes are Prominent Targets of B-cell Immunity

**DOI:** 10.1038/srep31657

**Published:** 2016-08-19

**Authors:** Franz J. J. Rieder, Julia Biebl, Marie-Theres Kastner, Martina Schneider, Christof Jungbauer, Monika Redlberger-Fritz, William J. Britt, Michael Kundi, Christoph Steininger

**Affiliations:** 1 Department of Medicine I, Div. of Infectious Diseases and Tropical Medicine, Medical University of Vienna, Vienna, Austria; 2 Blood Donation Center of the Austrian Red Cross for Vienna, Lower Austria and Burgenland, Vienna, Austria; 3 Department of Virology, Medical University of Vienna, Kinderspitalgasse 15, Vienna, Austria; 4 Department of Pediatrics, Children’s Hospital, Birmingham, USA; 5 Institute for Environmental Hygiene, Medical University of Vienna, Vienna, Austria

## Abstract

B-cell recognition of microbial antigens may be limited by masking of epitopes within three-dimensional structures (cryptotopes). Here we report that unmasking of cryptotopes by unfolding whole cytomegalovirus (CMV) antigen preparations with the chaotropic reagent Urea and probing with immune sera from healthy individuals (n = 109) increased ELISA signals by 36% in comparison to folded CMV antigens (P < 0.001). ELISA signals increased also significantly upon unfolding of *S. aureus* or *E. coli* antigens, whereas unfolded influenza H1N1 or respiratory syncitial virus antigens yielded reduced or unchanged reactivity in comparison to folded ones, respectively. Blocking of CMV cryptotope-specific Abs by incubation of an immunoglobuline preparation and three sera with unfolded CMV antigens enhanced clearly the neutralizing capacity of this immunoglobuline preparation against CMV infection. Thus, B-cell immunity frequently targets cryptotopes on CMV but these Abs are non-neutralizing, may reduce the neutralizing effectiveness of pathogen-specific Abs, and increase during immune maturation following primary CMV infection. The observation of functional consequences of Abs specific for cryptotopes may open whole new avenues to a better understanding of the humoral immune response to CMV and development of more effective vaccines and immunoglobuline preparations.

The primary B-cell response to an antigen (Ag) triggers a cascade of adaptation events that provides the immune system with antibodies (Ab) of high affinity and specificity. Adaptation requires several months and involves multiple positive and negative selection processes^1^. The vast pre-immune B-cell repertoire undergoes a stochastic process of positive selection upon first encounter with a specific infectious pathogen. The expansion of B-cell reactivity provides the host with a repertoire that is collectively capable of recognizing a nearly limitless array of pathogen-specific Ag determinants with highly variable affinities for Ag^2^. The limits of B-cell diversity are determined by accessibility of targets on the 3-dimensional structure of polypeptide Ags for ligation with the B-cell receptor (BCR). Hence, virtually the entire accessible surface of a protein represents an antigenic continuum^3^. Negative selection reduces pre-immune B-cell diversity by stringent scanning of BCRs for best available fit within a library of “imperfect” receptors to generate Ab of high specificity and affinity^4^,^5^.

Multiple microbial defence mechanisms, however, may counteract these adaptation mechanisms and allow microbes to establish chronic or latent infections. For example, processing and presentation of antigens by the MHC class I pathway may be subverted by microbial proteins^6^. Microbes may encode homologs of cellular cytokines/chemokines and their receptors to modify the hosts’ immune response^7^. Posttranslational modifications of microbial polypeptides, such as extensive glycosylation, may mask antigenic structures and thereby delay or even inhibit maturation of pathogen-specific B-cell immunity^8^,^9^,^10^.

Infectious pathogens may also counteract this process by masking potential immunological targets within their native three-dimensional structure (termed cryptic epitopes or cryptotopes)^8^,^9^,^10^. Cryptotopes may become immune targets occasionally upon protein unfolding^11^. Protein denaturation, proteolytic processing or presentation through Ag presenting cells (APCs) may expose hidden regions of a three-dimensional Ag to B-cell recognition^12^. Viral replication can involve structural plasticity of virions and temporal exposure of ostensibly cryptotopes^13^,^14^. Ligation to receptors on target cells may expose B-cell epitopes such as ligation of the HIV envelope glycoprotein 120 (gp120) to the CD4+ receptor^15^.

B-cell recognition of exposed microbial cryptotopes may block infectivity in selected instances^16^. More commonly, however, cryptotopes induce a subdominant, low-affinity, non-neutralizing Ab response, which may even interfere with the function of neutralizing Abs^17^,^18^. Neutralizing hepatitis C virus (HCV)-specific Igs, for instance, may become ineffective by non-neutralizing HCV Abs targeting an adjacent cryptotope^17^. Cross-reactive, non-neutralizing Abs generated during a primary Dengue virus infection against cryptotopes were proposed to enhance the pathogenicity of subsequent infections via the process of antibody-dependent enhancement^19^. Furthermore, the immunological net effect of B-cell responses to multiple conformational, linear, and cryptic epitopes of complex infectious pathogens is largely unknown.

We hypothesized that B-cell responses to microbial cryptotopes are common and that interference of Abs to conformational and cryptic epitopes may result in incomplete microbial neutralization. For a thorough evaluation of the net effect of the B-cell response to cryptotopes, we studied first Ab reactivity to conformational and cryptic epitopes of CMV. The CMV genome codes for >180 gene products of which >30 reportedly trigger a B-cell response^20^. Next, we validated our findings at the example of other common viral and bacterial pathogens. Finally, the neutralizing capacity of antibodies to these different CMV epitope classes was evaluated in a neutralization assay. We found evidence that the B-cell response to CMV but not to other viral pathogens is highly skewed towards recognition of cryptotopes and that Abs to CMV cryptotopes interfere with the function of neutralizing antibodies. This observation has potentially far reaching implication in the diagnosis, prevention, and treatment of a wide array of infectious diseases.

## Materials and Methods

### Clinical samples and antibodies

Human serum samples were collected within a prospective cohort study at the Medical University of Vienna, Austria (cohort I) and the Red Cross Vienna, Austria (cohort II). Written informed consent was obtained from all patients at the time of enrolment. The study protocol was approved by the local institutional review board of the Medical University of Vienna in accordance with the Declaration of Helsinki (EK26/2012). Cohort I included healthy volunteers (n = 18) and patients with primary CMV infection (n = 20). The diagnosis of primary CMV infection was established by the presence of CMV-specific IgM and CMV-DNA in plasma and clinical signs and symptoms compatible with infectious mononucleosis. None of the patients had a history of immunodeficiency or an other underlying disease. The median interval between onset of CMV disease and blood collection was 25 days, mean age of these patients was 40 years. Cohort II included healthy blood donors (n = 150). The mean age of these blood donors was 54 years (SD, 11 years; range, 24 to 74 years) and 88% (132 of 150) of these donors were male.

### Antigen preparations

The AD169 strain of CMV was propagated with the use of human foreskin fibroblasts (HFF), as previously described^21^. Cells were infected with CMV at a multiplicity of infection (MOI) of 0.01 and harvested as soon as a total cytopathogenic effect (CPE) developed. The propagation of the influenza A(H1N1)pdm09 strain was performed using MDCK-SIAT1 cells (HPA Culture Collection Cat.no. 05071502, passage number 6) according to standard procedures^22^. Respiratory syncitical virus (RSV) was propagated with the use of HeLa cells (ATCC^®^ CCL2^TM^, passage number 81) as described previously^23^.

Following collection of cell culture supernatant, residual cell debris was removed by centrifugation at 3000× g for 30 min. Concentration and further purification of virions was achieved by high-speed centrifugation at 33,000× g for 90 min. Viral pellets were resuspended in 1x Tris-buffered saline (TBS) (Bio-Rad, Hercules, CA, USA) containing 0.05% Tween-20 (TBS-T). To lyse virions, viral suspension samples were stored for 12 hours at 4 °C, sonicated, incubated again at 4 °C for 12 hours and stored at −20 °C^24^.

*Escherichia coli (E. coli*) strain JM109 was cultured in lysogeny broth (LB) for 12 hours at 37 °C. *Staphylococcus aureus* strain MRSA CCUG 26215 (agr1) (*S. aureus*) was cultured in Caso-Bouillon (Merck, Darmstadt, Germany) for 12 hours at 37 °C. To collect bacteria, samples were centrifuged (2500× g, 30 minutes, 4 °C) and bacterial pellets resuspended in 1x TBS-T followed by sonication for disruption of the cell membrane.

The protein concentration of bacterial and viral lysates was measured with use of the Quick Start Bradford Protein Assay Kit (Bio-Rad, Hercules, CA, USA) or BCA Protein Assay (Thermo Scientific, Waltham, Massachusetts, USA) according to the manufacturer’s instructions. Bovine serum albumin (BSA) was used as reference protein for quantification, concentrations ranging from 2 mg/ml to 0.125 mg/ml (Bio-Rad, Hercules, CA, USA).

### Recombinant expression of virus proteins

The open reading frames (ORFs) of CMV encoding the products of UL55 (glycoprotein B (gB)), UL32 (pp150 or pUL32), and UL83 (pp65) were cloned into the expression vector pcDNA 3.1. (Invitrogen Inc., Carlsbad, CA, USA). Human HEK293 cells were used for transient expression of all recombinant proteins following transfection using Lipofectamine 2000 (Invitrogen Inc., Carlsbad, CA, USA) according to the manufacturer’s recommendations. As a negative control, HEK293 cells were mock-transfected with the pCDNA 3.1. vector only. At 72 h post transfection, cells were harvested, washed with PBS and lysed by sonication. Subsequently, lysates were diluted in the appropriate buffer for enzyme-linked immunosorbent assay (ELISA).

### SDS-PAGE and immunoblotting

Transfected HEK 293 cells were harvested 72 hours post transfection, washed with PBS and lysed in Tris-buffer by sonication. Following protein measurement (BCA Protein Assay), samples were diluted with Tris-buffer and 5x reducing sample buffer (300 mM, 1 M Tris, pH 6.8, 50% glycerol, 0.05% Bromophenol blue, 10% SDS and 10% 2-Mercaptoethanol) and boiled at 99 °C for 5 minutes. Subsequently, sodium dodecyl sulfate polyacrylamide gel electrophoresis (SDS-PAGE) was performed with use of 4–15% gradient gels (Biorad, Hercules, CA, USA). After transfer to a PVDF membrane (#IPFL00010, Merck Millipore, Billerica, MA, USA), membranes were blocked with StartingBlock T20 (PBS) Blocking Buffer (Thermo Fisher Scientific, Waltham, MA, USA), incubated with an intravenous immunoglobulin preparations (IVIG), washed 3 times with PBS-T (0.05% Tween-20), incubated with anti-human secondary antibody (#sc2453, Santa Cruz, Dallas, TX, USA) and finally visualized using SuperSignal West Femto Chemiluminescent Substrate (Thermo Fisher Scientific, Waltham, MA, USA) and the ChemiDoc Imaging System (Biorad Inc., Hercules, CA, USA).

### Indirect ELISA

Viral, bacterial, and HEK293 lysates were diluted in the appropriate coating buffer, incubated for 12 hours at 4 °C and processed thereafter as described for each experiment. The following buffers were used as coating buffers in our ELISA experiments: Tris buffer (50 mM Trizma-base, 150 mM NaCl, 5% Glycerol), Urea buffer (8 M Urea, 100 mM NaH_2_PO_4_, 10 mM Trizma Base), Guanidin buffer (100 mM NaH_2_PO_4_, 10 mM Tris-HCl, 6 M Guanidin HCl). For a more permanent denaturation of antigens, Tris-treated viral lysates were precipitated with use of TCA (Trichloroacetic acid) and Urea-treated lysates with ethanol (U-E). For this purpose, lysate were incubated in TCA (final concentration, 12%) for 20 minutes, centrifuged for 10 minutes at 7,000× g, and pellets were subsequently washed with ice-cold acetone. Samples were then centrifuged for 10 minutes at 5,000× g, acetone was removed and pellets air-dried to remove residual acetone. Alternatively, viral lysates were treated with Urea-buffer and then precipitated by incubation in 100% ethanol (U-E) for 1 hour at −20 °C followed by centrifugation for 10 minutes at 10,000× g and removal of ethanol. Precipitates were resuspended in 1x Tris-buffered saline (TBS) and sonicated before use as antigen in the ELISA. All steps of the protein precipitation were done at 4 °C.

Microtiter plates (Corning Inc., Corning, NY, USA) were coated with clarified sonicates from viral or bacterial cultures, mock-transfected HEK293 cells or HEK293 cells transfected with vectors expressing either CMV gB, pUL32 or pp65 as described above. Following 12 hours of incubation at 4 °C, plates were washed four times with PBS with 0.05% Tween 20 (PBS-T) and then blocked with StartingBlock Blocking Buffer according to the manufacturer’s instructions (Thermo Fisher Scientific Inc., Waltham, MA, USA). Plates were incubated overnight (O/N) at 4 °C with primary Ab preparation diluted in blocking buffer. After washing the plates four times with PBS-T, plates were incubated with mouse anti-human, IgG-specific, horseradish peroxidase (HRP)-labeled secondary Ab (#ab97160, Abcam, Cambridge, UK) at a final dilution of 1:10,000 for 2 hours at room temperature. For labelling of mouse primary Abs, goat anti-mouse IgG-specific, HRP-labeled Ab (cat. no. sc2005, Santa Cruz Biotechnology, Inc., Heidelberg, Germany) at a final dilution of 1:1,000 was used. After washing the plates again four times with PBS-T, plates were incubated with TMB Microwell Peroxidase Substrate System (Kirkegaard& Perry Laboratories, Inc., Gaithersburg, MD, USA) for 20 minutes. The reaction was stopped with 1 *M* phosphoric acid and the optical density (OD) measured at a wavelength of 450 nm. Background signal was defined as mean OD without Ag.

To confirm equal amounts of protein adsorbed to ELISA plates despite the different treatment of antigen preparations, BSA was treated in parallel with Tris- or Urea-buffer and coated to the same ELISA plates as described for virus and cell lysates. Plates were washed thereafter four times with ddH_2_O instead of PBS-T to avoid interference of PBS-T with the silver staining solution used. Adsorbed proteins were visualized in the wells of the ELISA plates with use of a silver staining solution as described previously^25^. In brief, 100 μl of silver staining solution was added per well and plates were incubated for 100 minutes at room temperature. Subsequently, OD was measured at 405 nm and not corrected for readings at 620 nm as recommended^25^.

### Pre-adsorption of human sera for blocking neutralization assay

Immune sera in form of intravenous immunoglobulin preparation (IVIG) were heat-inactivated at 56 °C for 30 minutes before blocking of specific Ab fractions with use of native or denatured protein preparations at RT for 1 hour followed by 4 °C O/N. For the preparation of blocking antigens, CMV virions were lysed either in Tris- or Urea-buffer by two freeze-thaw cycles, sonication and incubation at 4 °C for 24 hours. The Urea-treated lysate was ethanol precipitated and resuspended with Tris-buffer to enable reliable protein measurement and storage conditions.

### Blocking neutralization assay

Evaluation of the neutralization capacity of human sera was done as described previously^26^,^27^. Briefly, immune sera with and without blocking antigens were serially diluted in media (DMEM supplemented with 5% calf serum), mixed with CMV strain AD169 and incubated for 60 min at 37 °C. Following incubation, 0.1 ml of virus-Ab containing mixture was added to confluent primary human dermal fibroblasts in 96-well microtiter plates and incubated for 60 min at 37 °C. The monolayers were washed twice with media and then incubated overnight at 37 °C. The monolayers were washed with PBS and then fixed in 100% ethanol for 10 min on the following day. Following washing with PBS, the monolayers were incubated with anti-IE-1 monoclonal Ab (#sc-69834, Santa Cruz, Dallas, TX, USA) for 1 h and binding developed with a FITC goat anti-mouse IgG (#sc2010, Santa Cruz, Dallas, TX, USA). Fluorescent nuclei were counted using an epi-fluorescence microscope adapted for reading 96 well plates. Results were presented as percent of inhibition calculated from maximal infectivity in controls.

### Statistical Analysis

Comparison of two groups was performed by use of the Mann-Whitney U test for quantitative parameters. Spearman-rho correlation coefficient was used to test for an association between non-normally distributed quantitative parameters. Dose-response curves from ELISA were assessed for parallelism by application of a four parameter logistic model. Fit was excellent with a pseudo R^2^ = 0.97. There was no significant deviation from parallelity and, therefore, relationship between preparations could be expressed as relative potency. Confidence intervals were computed based on Fieller’s theorem. A P-value < 0.05 was considered as statistically significant. All statistical tests were done with use of the software package SPSS 22.0.

## Results

### CMV-specific ELISA signals are enhanced by unfolding of viral antigens

**Figure 1:**
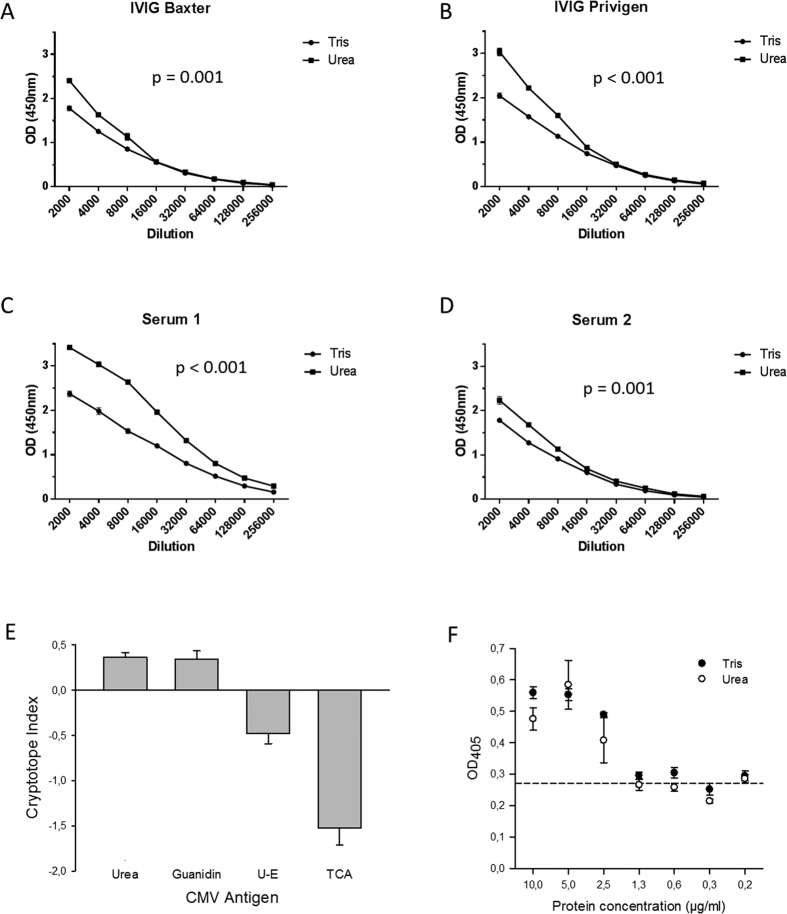
ELISA analysis of the reactivity of human polyclonal immune serum with native and denatured whole CMV lysate. (**A–D**) Representative OD_450_ readings obtained when probing native whole CMV lysates (Tris-buffer) or unfolded whole CMV lysates (Urea-buffer) with two-fold serial dilutions of immunoglobulin preparations (IVIG) and human polyclonal immune serum; error bars indicate standard error or the mean (SEM). (**E**) Mean OD_450_ readings obtained when probing native (Tris-buffer), unfolded (Urea- or Guanidin-buffer), or precipitated whole CMV lysate (ethanol (U-E) or TCA) with human polyclonal immune sera at a standard dilution (1:500; n = 18). Bars indicate mean CI measured and error bars indicate the standard error of the mean (SEM). (**F**) ELISA plates were coated with BSA preparations at different final concentrations, washed thoroughly, and stained with a silver stain to visualize protein adsorbed to the plates. The dashed line indicated mean OD_405_ readings measured for blanks. Optical density was measured at 405 nm and not corrected for reference measurements at 620 nm because silver absorbs light at both wave-lengths. All BSA experiments were done in triplicates.

Unfolding of polypeptide Ags may compromise discontinuous, conformational epitopes, leave linear epitopes unaffected, and unmask cryptotopes^28^. To evaluate the immunological net effect against CMV antigens, whole virus lysates were prepared under native and denaturing conditions and probed with immune sera from healthy individuals. First, proteins were chemically denatured with the chaotropic reagent Urea 8 M. ELISA signals obtained under these denaturing conditions were clearly higher than under native conditions and indicative for recruitment of additional, cryptotopes (Fig. 1A–D). The relative difference in signals was independent of the serum dilution used. For a uniform quantification of this difference in signals, we defined the cryptotope index (CI) in analogy to the avidity index^10^. The CI is calculated as the ratio of the optical density (OD) under native conditions to the OD_450_ under denaturing conditions (1-(ODNative/ODDenatured)). A positive index number indicates a high proportion of IgG with reactivity to cryptotopes. To validate the observed increase in signals by Urea treatment of lysates, experiments were repeated with use of a further chaotropic denaturant, Guanidin 6 M (Fig. 1E). The CI measured under the influence of Urea or Guanidin were similarly high and all experiments yielded the same difference in signals under native and denaturing conditions (median CI_Urea_, 0.36; SD_Urea_, 0.18 vs. median CI_Guanidin_, 0.31; SD_Guanidin_, 0.39). To exclude that the differences in measured OD_450_ may be attributed to differences in adsorption of protein preparations to ELISA plates because of antigen treatment, BSA was treated in parallel with Tris or Urea, adsorbed to the same ELISA plates, and adsorbed proteins were quantified with use of a silver staining procedure (Fig. 1F). Protein concentrations in coating solutions of 2.5 μg/ml yielded clearly positive results and OD_405_ readings measured did not differ significantly between these two protein preparations indicative of equal amounts of antigen bound to ELISA plates.

In addition, proteins were also denatured with use of Urea followed by ethanol-precipitation (U-E) or Tris-treated followed by TCA precipitation which reduces the propensity of protein refolding^29^. Signals obtained under these denaturing conditions were clearly lower than under native conditions and indicative for a reduced availability of CMV-specific epitopes (Fig. 1E). Remarkably, the harsher denaturing treatment of TCA precipitation also yielded a more pronounced decrease in ELISA signals in comparison to ethanol precipitation.

### Ab reactivity with CMV cryptotopes is associated with stage of infection

**Figure 2:**
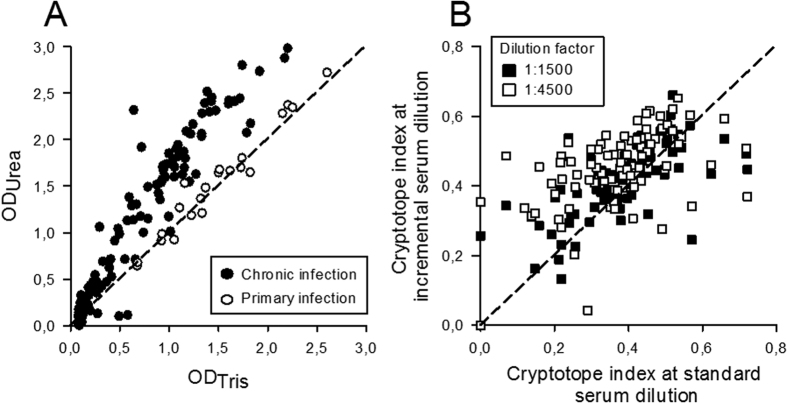
Evaluation of CI in healthy individuals. (**A**) Whole CMV lysates were probed under native (OD_Tris_) and denaturing (OD_Urea_) conditions with human immune sera from CMV-seropositive, healthy blood donors (n = 109) or from patients with primary CMV infection (n = 20). (**B**) Cryptotope index (CI) measured when testing human immune sera from CMV-seropositive, healthy blood donors (n = 109) at a standard serum dilution (1:500) in comparison to CI obtained when testing the same immune sera at higher dilutions (1:1500 and 1:4500, respectively). The dashed lines are references for similar signals obtained under native and denaturing conditions. Solid lines indicate linear regression plots for each group of patient samples.

The B-cell response to CMV antigens exhibits considerable inter-host variability^30^. To test whether this also holds true for reactivity with CMV cryptotopes, sera from a large cohort of unselected healthy blood donors (n = 150) were tested alike at a standard dilution (1:500) for CI. CMV-specific IgG were detectable in 73% (109 of 150) of the sera evaluated with use of the standard ELISA under native conditions. OD_450_ readings obtained under denaturing conditions correlated with those measured under native conditions (ρ = 0.95; P < 0.001 (Spearman-rho correlation coefficient)) and were overall clearly higher than those measured under native conditions (median CI 0.36; IQR, 0.21–0.44; Fig. 2A). Neither age nor sex of the present blood donors was associated with CMV-specific CI (P = 0.556 and P = 0.767 (Mann-Whitney U)).

Optical density measured in ELISA is a three-parameter logistic function of Ig-concentration and may yield false positive results in sera with low Ab titres and vice versa^10^. To evaluate whether concentration of CMV-specific Abs influences also CI, the CMV immune sera (n = 109) were tested in parallel at two further dilutions (1:1500 and 1:4500). This analysis showed that the CI measured at the standard dilution (1:500) correlated well with the CI measured at the 1:1500 and 1:4500 serum dilutions (P < 0.001, respectively (Spearman-rho correlation coefficient), Fig. 2B).

To evaluate whether CMV-specific CI is associated with the stage of CMV infection, sera from patients with primary CMV infection (n = 20) were also evaluated. Inter-host variability of CI was also low for samples from patients with primary CMV infection (median CI, 0.03; IQR, −0.02–0.07; Fig. 2A). Remarkably, the CI obtained with these samples was clearly lower in comparison to probing CMV lysates with immune sera from healthy blood donors indicative for a process of immune maturation (Fig. 2A).

### Cryptotopes on individual CMV Ags

**Figure 3:**
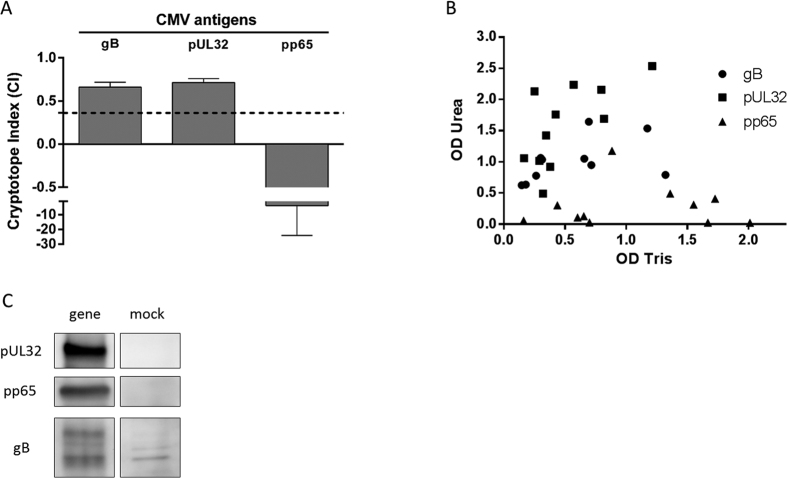
Evaluation of CI against different CMV Ags. The highly immunogenic CMV Ags glycoprotein B (gB, UL55), pUL32 (pp150), and major structural protein pp65 (UL83) were probed with use of human CMV immune sera (n = 11) under native (Tris-buffer) and denaturing (Urea-buffer) conditions. (**A**) The difference in signals obtained for each serum sample was expressed as cryptotope index (CI). Bars indicate median CI measured and error bars indicate the interquartile range (IQR). Dashed line indicates median CI of whole CMV (n = 109). (**B**) Same data set expressed as scatterplot indicating OD_Tris_ and OD_Urea_ of each serum for the respective antigen. (**C**) Western blot imaging of HEK 293 cells transfected with pUL32-, pp65- and gB (gene) and mock-transfected, probed with IVIG.

CMV whole virus lysate comprises a complex assortment of >30 B-cell Ags, which additionally harbour multiple conformational, linear, and very likely, cryptotopes^20^. To elucidate the relative contribution of individual CMV antigens to the overall B-cell response to CMV cryptotopes, three of the most immunogenic CMV antigens (gB, pp65, pUL32) were evaluated also under native (Tris) and denaturing (Urea) conditions. Of the 16 sera from healthy controls, 11 reacted with all three antigens. The evaluation using these sera revealed positive CI for gB and pUL32 (median CI 0.66 and 0.71, respectively) and a clearly negative CI for pp65 (median CI −3.94) (Fig. 3A,B). Western blot imaging confirmed expression of gB, pp65, and pUL32 by the transfected mammalian cells (Fig. 3C).

### Ab reactivity with cryptotopes is associated with infectious pathogen

**Figure 4:**
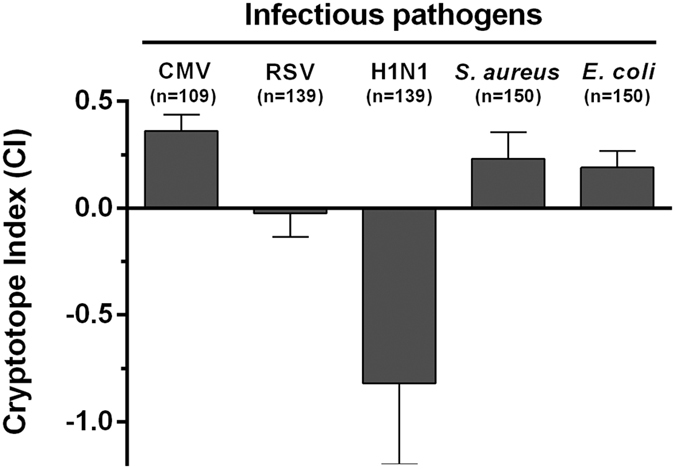
Analysis of cryptotope indices for different viral and bacterial pathogens. Human immune sera were probed for reactivity with common infectious pathogens (CMV, human Cytomegalovirus (n = 109); RSV, respiratory syncytial virus (n = 139); H1N1, influenza virus strain H1N1 (n = 139); *S. aureus, Staphylococcus aureus* strain MRSA CCUG 26215 (agr1) (n = 150); *E. coli, Escherichia coli strain* JM109 (n = 150)) under native (Tris) and denaturing (Urea) conditions. Bars indicate the median CI of all sera evaluated and error bars indicate the IQR. Volumes of serum samples were not sufficient in 11 patients to be tested also in the RSV and H1N1 assay.

To test whether the variability in CI observed for the different CMV Ags reflects also differences between various human infectious pathogens, CI was determined also for reactivity of immune sera against two common bacterial and viral pathogens (*Escherichia coli* strain JM109, *Staphylococcus aureus* strain CCUG 26215, Influenza virus strain H1N1 and respiratory syncitial virus (RSV)), respectively. Probing the bacterial Ag preparations with the immune sera under native and denaturing conditions yielded also positive CI (median CI *E. coli*: 0.190, *S. aureus*: 0.232) similarly to CMV whole virus lysate (median CI 0.36) (Fig. 4). In contrast, the CI measured for the influenza H1N1 was clearly negative (median CI −0.819) and for RSV neutral (median CI −0.024) which indicates a lower frequency of B-cells targeting cryptotopes than in CMV infection.

### Functional consequences of Ab reactivity with cryptotopes

**Figure 5:**
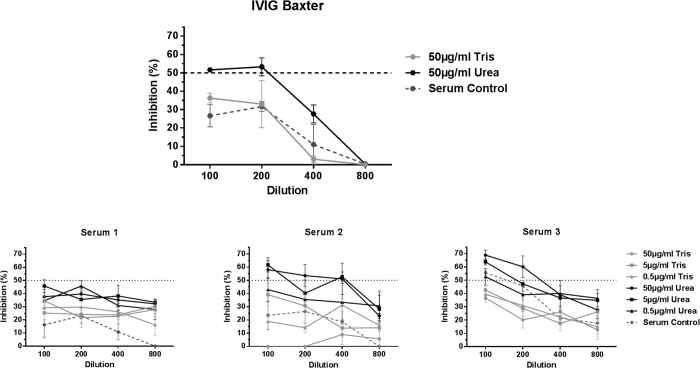
Evaluation of neutralizing efficacy of immune sera from one patient with recent primary infection (serum 1) and two patients with latent infection (serum 2 & 3) and an immunoglobulin preparation (IVIG Baxter) after blocking of different fractions of CMV-specific Igs. Complement-inactivated sera and IVIG was either diluted with Tris-buffer (control) or pre-incubated with native whole CMV lysate (Tris) to block conformational and linear epitopes or unfolded whole CMV lysate (Urea-treated, precipitated with ethanol) to block preferentially cryptotopes. The horizontal, dashed line indicates 50% neutralization of virus. Final viral protein concentrations used for the IVIG experiment was 50 μg/ml and 50, 5 and 0.5 μg/ml for the experiments with the sera. Final dilution ranged from 1:100–1:800 as indicated in the figure. All experiments were done in triplicates and error bars indicate the standard error of the mean.

In order to evaluate the functional consequences of Ab ligation to CMV cryptotopes, we tested the neutralizing capacity of immune sera from one patient with recent primary infection (serum 1) and two patients with latent infection (serum 2 & 3) and an immunoglobulin preparation (IVIG Baxter) with and without blocking of the different Ab fractions with use of native or unfolded CMV lysate (Fig. 5). IVIG are comprised of a complex amalgam of polyclonal Abs pooled from a large number of healthy plasma donors without modifications to the composition of Igs and therefore reflect the serostatus of the general population. All sera and the IVIG neutralized CMV in a dose-dependent manner (Fig. 5). Virus neutralization was higher with the sera from the latently infected individuals than from the recently infected one and the IVIG. Blocking of serum Abs with Urea-treated CMV lysate improved neutralization in all four serum preparations, whereas blocking with Tris-treated CMV lysate reduced the neutralization capacity.

## Discussion

The B-cell response to infectious pathogens is directed against an array of conformational and linear epitopes on the exposed, three-dimensional structure of the microbe. The B-cell response to cryptotopes, however, may also be of high immunological relevance but did not receive much attention so far. Only a small number of cryptotopes have been identified so far on flaviviruses, HCV, HIV, *Plasmodium falciparum* and influenza viruses^13^,^15^,^31^,^32^,^33^. Our studies, however, indicate a high abundance of cryptotopes on selected viral and bacterial pathogen and an interference of this Ab fraction with neutralizing Ig. Recognition of B-cell responses to cryptotopes may have far reaching implications for the diagnosis, prevention and treatment of infectious diseases.

Urea potently denatures protein as a function of time, temperature and molarity^34^. We used urea at 8 M to denature the presently evaluated whole microbial lysates and recombinant proteins. Nevertheless, refolding of proteins may occur rapidly upon removal of the denaturant urea and may be expected also in our experiments as plates were washed thoroughly with PBS-T before incubation with Abs to avoid interference of Urea with Ab binding^34^. Still, we found clear differences in signals when using ELISA in different formats compatible with only partial refolding of Ags upon removal of the denaturants and conserved exposure of a large fraction of cryptotopes to ligation with specific Igs. These observations indicate in concordance with previous studies that destabilization of folded protein structures is sufficient to expose cryptotopes^35^. Moreover, refolding of denatured proteins may have been incomplete due to the hydrophobic polystyrene surfaces of ELISA plates used and folding and assembly of polypeptides *in vivo* requires other, chaperon proteins^36^.

Hypothetically, treatment of proteins with denaturing reagents may change binding strength to ELISA plates in comparison to proteins coated under native conditions. To evaluate whether equal amounts of protein were adsorbed to ELISA plates, we quantified BSA adsorbed to ELISA plates after treatment with Tris- or Urea-buffer with use of silver staining protein. Coating of protein was similarly efficient for Tris- and Urea-treated proteins although somewhat lower for Urea-treated proteins at the protein concentration used in the ELISA (2.5 μg/ml). Nevertheless, ELISA signals were higher for Urea-treated antigens and binding effectiveness may therefore not be explained by differences in protein coating. In addition, testing of H1N1 or RSV lysates yielded a negative CI which indicates further that the higher ELISA signals measured for CMV, *S. aureus*, or *E. coli* lysates may not be attributed to higher protein quantities adsorbed to ELISA plates.

In viral infection, the most important function of Abs is neutralization of infectivity by binding to viral surface components and thereby interfering with functions that are essential for virus entry into cells^37^. Cryptotope-specific Abs are assumed to be ineffective during most stages of viral replication and are mostly subdominant and non-neutralizing^33^. Still, our observations indicate that (i) the healthy human immune system invests considerable resources into the B-cell response to CMV cryptotopes, (ii) the prominent B-cell response to cryptotopes separates CMV from infection with other viral pathogens, and (iii) the relative fraction of cryptotope-specific Abs appears to increase with time after primary infection. Taken together, it may speculated that skewing of CMV-specific B-cell immunity towards cryptotopes adds a further mechanism of CMV immune modulation and evasion to the already >100 mechanisms described so far^38^.

Infectious pathogens commonly evade the immune response by hiding structures of importance for replication and infection^16^,^17^,^39^. pUL32, for instance, is the large tegument protein of CMV, indispensable for CMV replication^40^, hidden within the virion and still induces a strong, non-neutralizing B-cell response with an additional prominent targeting of cryptotopes^41^,^42^. Partial disintegration of mature or immature viral and subviral particles, secretion of soluble forms of viral proteins from infected cells, release of incompletely processed and/or assembled viral proteins from lysed cells, as well as structural plasticity of virions may expose cryptotopes during the course of infection^33^. Accumulation of such events during maturation of CMV-specific immunity may gradually shift the focus of B-cell immunity from neutralizing conformational to non-neutralizing cryptic epitopes and prolong Ab avidity maturation^10^. On the other hand, pUL32-specific Abs are rarely boosted by CMV reactivation which is indicative for an infrequent presentation during reactivations from latency^43^. Hence, the detection of cryptotope-specific Abs does not inevitably indicate biological or immunological significance for microbe or host.

The net effect of Ab interactions measured *in vitro* may not necessarily be the same *in vivo*. For example, gB is the CMV antigen most commonly used in subunit vaccines because of a robust induction of neutralizing Igs. Multiple distinct B-cell epitopes were identified on gB that may induce neutralizing as well as non-neutralizing Abs^44^. Our studies indicate the additional presence of gB cryptotopes that react with a large fraction of gB-specific Igs. The competition of neutralizing and non-neutralizing gB-specific Abs observed presently for whole CMV lysate or previously for HCMV^44^ stands in contrast to the protective B-cell immunity induced in a recent CMV vaccine trial with use of a full-length gB protein Ag^45^. On the other hand, immunity induced with this gB vaccine was short-lived in concordance with our observed shift from targeting of conformational to cryptic epitopes during immune maturation. Selection of Ags according to propensity to induce a B-cell response to cryptotopes may improve significantly long-term efficacy of vaccines.

Treatment of patients with IVIG may prevent or mitigate a variety of infectious diseases (reviewed in ref. 46). IVIG are comprised of a complex amalgam of polyclonal Abs pooled from a large number of healthy plasma donors without modifications to the composition of Abs. The efficacy of CMV IVIG preparations used for the prevention of CMV infection or disease did not reach statistical significance in clinical trials^46^,^47^,^48^. In contrast, measles virus, influenza virus or RSV infection may be efficiently prevented with use of IVIG preparations^49^,^50^,^51^. Cryptotopes may be found on CMV as well as on influenza viruses^18^. Our studies, however, indicate that Abs against CMV cryptotopes constitute a significantly larger fraction of virus-specific Ig in IVIG than those against influenza or RSV. Moreover, we could improve significantly the neutralizing capacity of sera and IVIG by blocking of Abs against CMV cryptotopes which apparently interfered with the function of neutralizing Igs. Apparently, Abs against cryptotopes may reduce the efficacy of IVIG in a dose-dependent manner and complete blocking of all CMV epitopes was not required to observe this effect as neutralization was not reduced significantly when using native CMV lysate for blocking. Although results have to be interpreted with caution in view of the limited number of samples tested and the use of only epithelial and not endothelial cells for viral propagation, removal of non-neutralizing fractions of CMV-specific Abs has the potential to improve clearly the effectiveness of IVIG in the prevention and treatment of CMV disease.

In conclusion, we identified a strong B-cell immune response to cryptotopes of CMV and other bacterial pathogens. The relative abundance of Ig fractions against cryptotopes increases with the maturation of immunity following primary CMV infection. The observation of functional consequences of Abs specific for cryptotopes may open whole new avenues to a better understanding of the humoral immune response and developing significantly more effective IVIG for the treatment of an array of infectious diseases.

## Additional Information

**How to cite this article**: Rieder, F. J. J. *et al*. Microbial Cryptotopes are Prominent Targets of B-cell Immunity. *Sci. Rep.* **6**, 31657; doi: 10.1038/srep31657 (2016).
